# Obituary

**Published:** 1860-07

**Authors:** 


### OBITUARY.

Died, at Louisville, Ky., June 6, Mr. ORANGE SAYLOR, of Rheumatic Carditis.

Mr. Saylor was a student of medicine, and attended the last session of Rush Medical College. He had been for many years a sufferer from rheumatism, which resulted in hypertrophy of the heart. Last winter, while engaged as prosector to the Professor of Anatomy, he was severely attacked with his old disease, disabling him entirely from his duties. He had several renewals of the attack through the spring, and had started south in hopes a milder climate might relax the grasp of the fell torturer. He reached Memphis, where he found himself so severely affected he could go no farther, started to return, got as far as Louisville, where he breathed his last.

It will be with many and sincere regrets that this notice will be read by his classmates and friends. He was a young man of quick perception, great energy and industry, and by his generous frankness and manly bearing endeared himself to both teacher and student.

He was most enthusiastically attached to his adopted profession, and we feel that his progress last winter gave earnest of a pre-eminent professional career.

☞ That excellent publication, the *New York Journal of Medicine*, after having existed for seventeen years without change, has finally submitted to the law of mutation incident to all earthly things; and henceforth will be issued weekly, under the name of the *American Medical Times*. Each number will consist of 24 quarto pages, double columns, filled with such general and scientific matter as may be of interest to the profession.

